# 

**Published:** 1889-11

**Authors:** 


### LITERARY.

WE HAVE received from the Chancellor of the new National University of Chicago the "Announcement" for 1889-90, a neat publication of sixty-four pages, which gives a list of forty non-resident professors who are well-known scholars, many of them being professors of such institutions as the University of Virginia, Tulane, Boston, Madison and Lehigh Universities.

The Announcement gives thirteen under-graduate courses and an equal number of post-graduate courses leading to all the various college degrees. The institution is said to be modelled after the famous London University and provides examinations whereby scholars can secure degrees by non-resident study and examinations at home, thus benefiting a large class and solving many educational problems. It also agrees to teach "any person in any subject" by mail, and expects to introduce here the University Extension System of Great Britain by which local lectures are given by the professors, thus bringing the University closer to the people.

No honorary degrees will be given. The tuition is moderate, and the University affiliates with all other institutions by accepting their certificates. Two hundred students are said to be already enrolled. The Announcement will be sent free to any one who addresses 147 Throop Street, and our readers who apply before November 10th need not pay the usual matriculation fee.

THE CENTURY MAGAZINE enters upon its twentieth year the present month. Among its leading articles are the opening pages of the autobiography of the famous actor, Joseph Jefferson, so long identified with the character of Rip Van Winkle, as presented at our principal theatres. Everybody will want to read it.

THE MANIFESTO—organ of the United Societies of Shakers. This excellent monthly entered upon its XIXth Volume in September, and comes to us greatly improved in style.

THE SHELTERING ARMS.—The 25th annual report of this worthy and prosperous institution for the care of homeless children, shows that during the past twenty-five years 1,822 children have received the benefit of its protecting care.

STUDIES IN INTESTINAL SURGERY, by Wm. B. Van Lennep, A. M., M. D., a treatise of value to the medical profession, has been laid upon our table.

THE DISPOSAL OF THE DEAD, by J. M. Peacock, M. D., is a historical and timely essay, giving much valuable information upon a subject largely occupying public attention.

---

### SALEM BY THE SEA.

In the olden time 'twas deemed a crime—
So said the law—so says my rhyme—
For maid or wife to vex ones life
By witcheries of antics rife;
And fiercest then her bounds to free,
Was Salem—Salem by the sea.

But Salem now is glad t'allow
Her witching maids of thoughtful brow,
And graces rare, to beguile us there,
To building castles in the air;
Whilst Salem smiles complacently,
Oh Salem! Salem by the sea!

